# Cancer Immunotherapeutic Potential of NKTT320, a Novel, Invariant, Natural Killer T Cell-Activating, Humanized Monoclonal Antibody

**DOI:** 10.3390/ijms21124317

**Published:** 2020-06-17

**Authors:** Nishant P. Patel, Peng Guan, Devika Bahal, Tanwir Hashem, Felix Scheuplein, Robert Schaub, Kim E. Nichols, Rupali Das

**Affiliations:** 1 Division of Oncology, Children’s Hospital of Philadelphia, Philadelphia, PA 19104, USA; patelnishantp@yahoo.com (N.P.P.); guanp@email.chop.edu (P.G.); 2 Department of Physiology, Michigan State University, East Lansing, MI 48824, USA; bahaldev@msu.edu (D.B.); hashemta@msu.edu (T.H.); 3 Immuno-Oncology, Blueprint Medicines, Cambridge, MA 02139, USA; felix.scheuplein@gmail.com; 4 RGS Consulting, 118 Jeremy Hill Road Pelham, Pelham, NH 03076, USA; rgschaub@aol.com; 5 Department of Oncology, St. Jude Children’s Research Hospital, Memphis, TN 38105, USA; kim.nichols@stjude.org

**Keywords:** antitumor immunity, monoclonal antibody, NKTT320, invariant natural killer T cells, immunotherapy

## Abstract

Invariant natural killer T cells (iNKTs) directly kill tumor cells and trans-activate the anti-tumor functions of dendritic cells (DC), natural killer (NK) cells, and T and B cells. As such, iNKTs serve as a powerful tool for use in cell-based cancer immunotherapy. iNKT cell activation commonly requires engagement of the invariant T cell receptor (iTCR) by CD1d presenting glycolipid antigens. However, transformed cells often down-regulate CD1d expression, which results in a reduction of iNKT cell anti-tumor functions. One approach to circumvent this critical barrier to iNKT cell activation is to develop an agonistic antibody that binds directly to the iTCR without the requirement for CD1d-mediated antigen presentation. To this end, we have characterized the iNKT cell stimulatory properties of NKTT320, a novel, recombinant, humanized, monoclonal antibody that binds selectively and with high affinity to human iTCRs. Strikingly, immobilized NKTT320 mediated robust iNKT cell activation (upregulation of CD25 and CD69) and proliferation (carboxyfluorescein succinimidyl ester (CFSE) dilution), as well as Th1 and Th2 cytokine production. Additionally, iNKTs stimulated by plate-bound NKTT320 exhibited increased intracellular levels of granzyme B and degranulation (exposure of CD107 on the cell surface). Furthermore, both soluble and immobilized NKTT320 induced iNKT cell-mediated activation of bystander immune cells, suggesting that this novel anti-iTCR antibody facilitates both direct and indirect iNKT cell cytotoxicity. These studies are significant, as they provide a framework by which iNKT cell anti-cancer functions could be enhanced for therapeutic purposes.

## 1. Introduction

Invariant natural killer T cells (iNKTs) are unique, innate-type T lymphocytes that develop in the thymus and have characteristics of both conventional T cells and natural killer (NK) cells [1]. Human iNKTs express a highly conserved invariant T cell receptor (iTCR) that consists of the Vα24-Jα18 chain joined to the Vβ11 chain, while mouse iNKTs express a Vα14-Jα18 chain that pairs preferentially with Vβ2, Vβ7, or Vβ8.2 chains [2]. Unlike conventional T cells that recognize peptide antigens presented in complexes with major histocompatibility complex (MHC) molecules, iNKTs recognize glycolipid antigens, such as the marine-derived glycolipid, α-galactosylceramide (αGC), or its synthetic analogue KRN7000, when presented by the MHC-like protein CD1d [1,2]. Following iTCR stimulation, iNKTs rapidly produce multiple cytokines and chemokines, and induce the expression of co-stimulatory molecules on antigen-presenting cells (APCs). As a result, they have the capacity to modulate both the innate and the adaptive immune responses [3], and thus direct the immune response during infection, inflammation, and cancer [4].

Reports of human patients suggest an active role for iNKTs in protection against tumors. For example, patients with various types of cancer have reduced iNKT cell frequencies and functional responses [5,6,7,8]. Conversely, in patients with neuroblastoma [9], colorectal [10], and head and neck carcinomas [11], increased numbers of circulating iNKTs correlate with better clinical outcomes and prolonged survival. Similarly, studies in mouse models demonstrate that iNKT-deficient mice are more susceptible to tumors [12,13], whereas the reconstitution of iNKTs reduces or prevents tumor progression [14]. Invariant NKTs mediate their anti-tumor effects via various mechanisms, including direct cytotoxicity against CD1d+ tumor cells [15,16,17] or tumor-associated macrophages [18]. Additionally, iNKTs transactivate other immune cells, such as the T, B, NK, and dendritic cells (DCs), and augment their anti-tumor functions [19,20]. Thus, iNKTs hold great potential for use as a cell-based cancer therapy. However, tumor cells often downregulate CD1d, and thereby evade iNKT cell recognition [21,22,23]. To overcome this critical barrier, several novel approaches, including bi-specific fusion proteins and chimeric antigen receptor (CAR)-transduced iNKTs, have recently been tested in preclinical tumor models, where they have exhibited significant anti-tumor activity [24,25,26]. However, these approaches are limited by the number of known tumor antigens against which iNKTs may be targeted. 

Recently, NKT14m, a monoclonal antibody (mAb) specific to murine iTCRs, was reported to activate murine iNKTs in vivo [27] and promote iNKT cell anti-tumor activity in a murine model of B-cell lymphoma [28]. These data suggest that a similar iNKT-activating antibody could provide a novel means to treat human cancer. In the current study, we characterized NKTT320, a humanized monoclonal antibody directed against the human iTCR. Our data demonstrate that soluble and immobilized NKTT320 induce dose-dependent human iNKT cell activation, cytokine production, proliferation, degranulation, and activation of bystander immune cells. Taken together, these data support further development of this or other iTCR engaging antibodies as a novel means of capitalizing on the anti-tumor activities of iNKTs in the treatment of human cancers. 

## 2. Materials and Methods

### 2.1. Reagents

Alpha-galactosylceramide (αGC; KRN7000) was purchased from Enzo Life Sciences (Farmingdale, NY, USA). Recombinant human (rh) interleukin (IL)-2 and IL-15 were purchased from Peprotech (Rocky Hill, NJ, USA) and Sigma (St. Louis, MO, USA), respectively. Carboxyfluorescein succinimidyl ester (CFSE) and the MILLIPLEX kits were purchased from Molecular Probes, Invitrogen (Grand Island, NY, USA) and Millipore (Billerica, MA, USA), respectively. PBS57-loaded human CD1d tetramers were obtained from the NIH Tetramer Core Facility (Atlanta, GA, USA). NKTT320 antibody was provided by NKT Therapeutics. NKTT320 is an IgG4 mutated to stabilize heavy chain dimer formation and ablate residual Fc receptor binding activity [29].

### 2.2. Flow Cytometry

Monoclonal antibodies used for human NKT cell staining included Vα24 and Vβ11 (Beckman Coulter; Brea, CA, USA); CD3, CD4, CD56, CD69, CD19, and CD11c (BD PharMingen, San Jose, CA, USA); CD25, CD107a, and FasL (eBiosciences; San Diego, CA, USA); CD80, CD86, HLA-DR-DP, and TRAIL (BioLegend; San Diego, CA, USA). Intracellular perforin and granzyme B were detected using antibody from BioLegend and Invitrogen (Grand Island, NY, USA), respectively. Data was collected on a BD LSRII flow cytometer (BD Biosciences, San Jose, CA, USA) and analyzed using FlowJo software (FlowJo LLC; Ashland, OR, USA). 

### 2.3. Human iNKT Cell Expansion and Purification

Peripheral blood mononuclear cells (PBMCs) were obtained from the Immunology Core Facility at the University of Pennsylvania (Philadelphia, PA, USA) or Zenbio (Research Triangle Park, NC, USA). PBMCs were cultured in complete AIM-V media (Gibco, Waltham, MA, USA) with 10% fetal bovine serum (FBS), KRN7000 (αGC) at 500 ng/mL, and recombinant human (rh) IL-2 (Peprotech, Rocky Hill, NJ, USA) at 50 U/mL. On the fourth day, rhIL-15 (Peprotech) and rhIL-2 were added at 10 ng/mL and 10 U/mL, respectively, to the culture. After four more days, human iNKT cells were purified by staining with FITC-conjugated, anti-Vα24 antibodies, followed by anti-FITC magnetic beads, per manufacturer’s instructions (Miltenyi Biotech, Auburn, CA, USA). Post-sort FACS analysis revealed >95% PBS57–CD1d tetramer reactive cells.

### 2.4. Statistics

Statistical analyses were performed using GraphPad PRISM software (San Diego, CA, USA). A *p*-value less than or equal to 0.05 was deemed to be significant.

## 3. Results

### 3.1. NKTT320 Induces Human iNKT Cell Activation and Proliferation 

**Figure 1:**
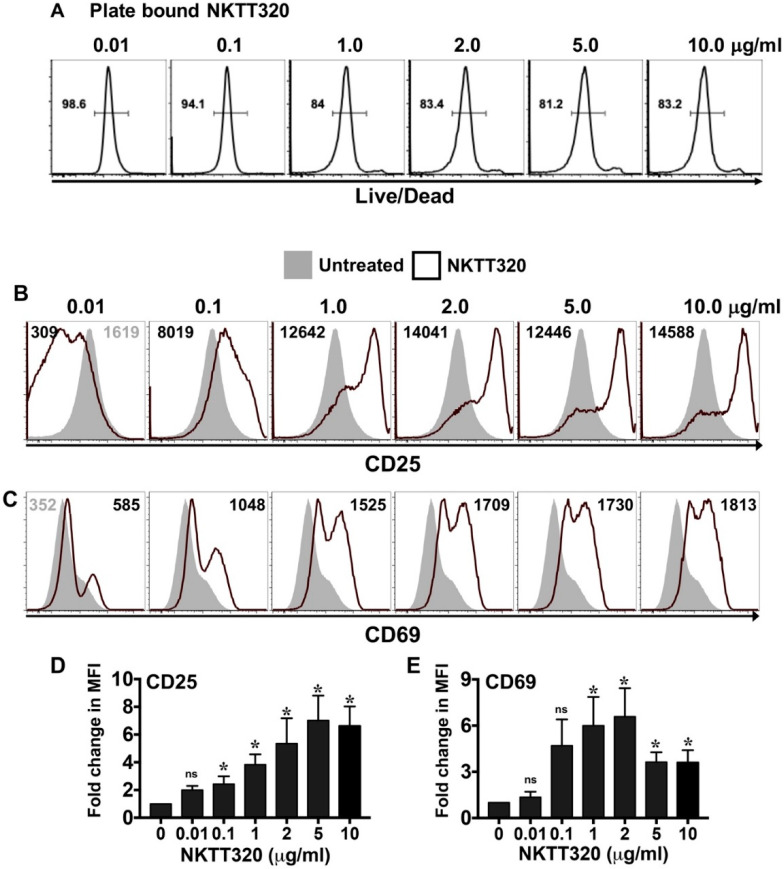
Dose-dependent activation of human invariant natural killer T cells (iNKTs) in the presence of immobilized NKTT320 in vitro. (**A**–**E**) Human iNKTs were incubated with no stimulus or increasing concentrations of plate-bound NKTT320 mAb. After 24 h, cells were stained with LIVE/DEAD Fixable Aqua Dead Cell dye and analyzed for viability (**A**) by flow cytometry. Representative histograms from one of six independent experiments are shown. Numbers in the histograms indicate percent viable cells. (**B**–**E**) Cells were also analyzed for expression of the activation markers CD25 (**B**) and CD69 (**C**) by flow cytometry. Representative histograms from one of five independent experiments are shown. Numbers in the histograms indicate the mean fluorescence intensity (MFI). Compiled data (mean ± SEM) from five independent experiments show fold increases in CD25 (**D**) and CD69 (**E**) expression on iNKTs plated on immobilized NKTT320, as compared to cells left untreated. Statistical significance in (**D**,**E**) was determined using an unpaired *t*-test with Welch’s correction. For each of the analysis, all the groups were compared to cells that were left unstimulated. * *p* < 0.05, ns: not significant.

**Figure 2:**
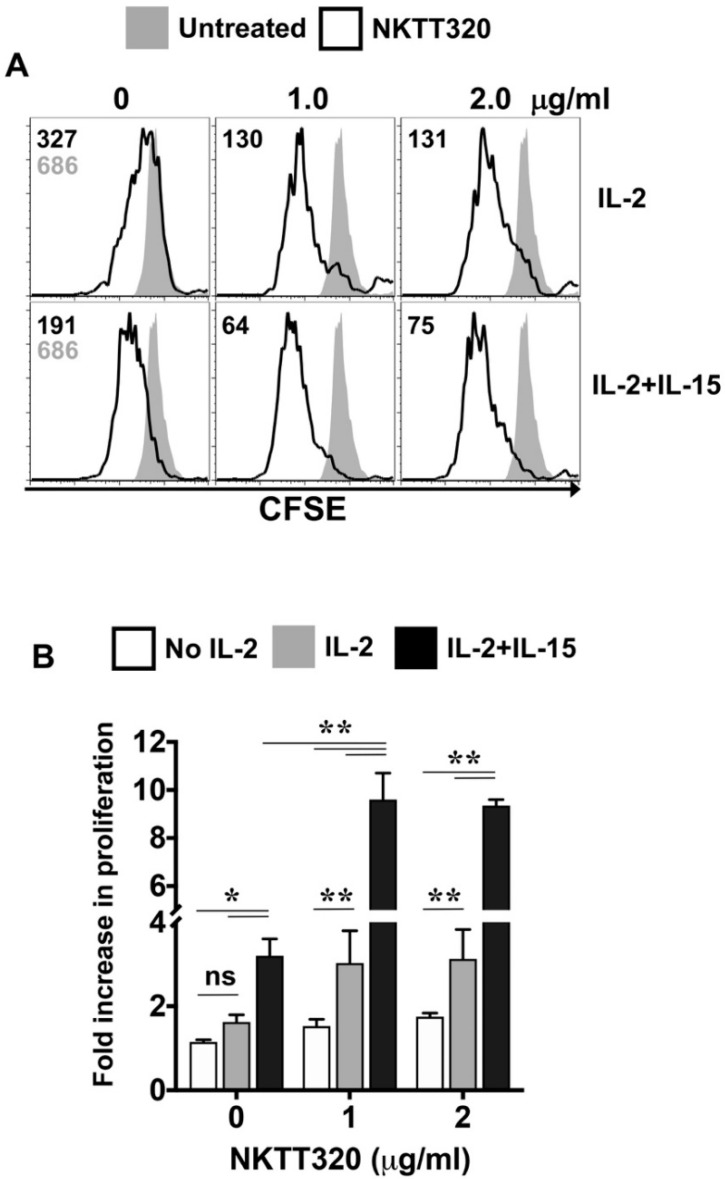
Plate-bound NKTT320 induces human iNKT cell proliferation in vitro. (**A**,**B**) Human iNKTs were labeled with 250 nM of carboxyfluorescein succinimidyl ester (CFSE) on day 0 and then stimulated with different concentrations of plate-bound NKTT320 mAb in the presence of low-dose interleukin (IL)-2 (10 μg/mL) or IL-2 + IL-15 (50 μg/mL), as indicated. After four days, cells were harvested and analyzed for cell proliferation by flow cytometry. (**A**) Representative histograms from one of three independent experiments are shown, and numbers in the histograms indicate MFI. (**B**) Fold increase in proliferation was calculated as a ratio of the MFI of cells left untreated for 96 h over the MFI of iNKTs cultured on plate-bound NKTT320 mAb with or without IL-2 and IL-15. Pooled data (mean ± SEM) from three independent experiments are shown. Significance in (**B**) was determined by two-way ANOVA with Tukey’s multiple comparison. * *p* < 0.05, ** *p* < 0.01, ns: not significant.

To test whether NKTT320 can activate human iNKTs, we cultured purified human iNKTs on varying concentrations of plate-bound, NKTT320 monoclonal antibodies (mAb). Important for our studies, the viability of these cells was maintained at more than 80%, even at the highest concentration of the NKTT320 tested (Figure 1A). Incubation of iNKTs with varying concentrations of the immobilized mAb increased the expression of activation markers CD25 and CD69, with an average of 4–6-fold increases in the mean fluorescence intensity (MFI). Responses were dose-dependent and occurred at concentrations as low as 0.1 μg/mL (Figure 1B–E). We observed a similar increase in the expression of CD25 and CD69 in the presence of soluble NKTT320 (data not shown). To assess whether NKTT320 promotes iNKT cell proliferation, experiments were performed using CFSE-labeled iNKTs. We observed some basal proliferation of iNKTs (demonstrated by the dilution of CFSE) in the presence of IL-2 alone (1.625 ± 0.17-fold), which was increased in the presence of IL-15 (3.2 ± 0.40-fold). However, this cytokine-induced iNKT cell proliferation was significantly upregulated in the presence of plate-bound NKTT320 (Figure 2A,B). The fold increase in proliferation of iNKTs at 1.0 μg/mL of mAb were 1.525 ± 0.17 (no IL-2), 3.025 ± 0.77 (+IL-2), and 9.6 ± 1.1 (+IL-2 + IL-15).

### 3.2. NKTT320 Stimulates Robust Human iNKT Cell Cytokine Production In Vitro

**Figure 3:**
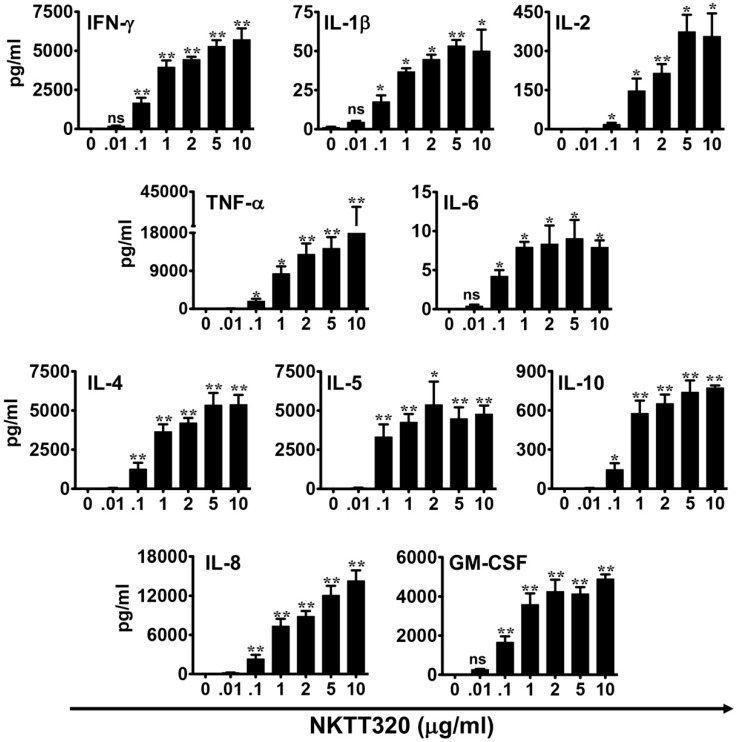
NKTT320 promotes Th1/Th2 cytokine production by human iNKTs. Human iNKTs were incubated with increasing concentrations of plate-bound NKTT320 mAb. After 24 h, cell culture supernatants were harvested and analyzed for Th-1 and Th-2 cytokines by Luminex. Data is presented as mean ± SD from one of two independent experiments. Statistical significance was determined using an unpaired *t*-test with Welch’s correction. * *p* < 0.05, ** *p* < 0.01, ns: not significant.

Invariant NKT cells contribute to host immunity against tumors, largely by rapid and robust production of both Th1 and Th2 cytokines [3]. Therefore, we next determined the ability of NKTT320 to induce cytokine production by iNKTs. Purified human iNKTs were plated overnight on varying concentrations of immobilized NKTT320. Analysis of culture supernatants revealed that the mAb promoted the abundant secretion of numerous cytokines, including interferon-gamma (IFN-γ), tumor necrosis factor-alpha (TNF-α), granulocyte macrophage colony stimulating factor (GM-CSF), and interleukins (ILs)-2, 4, 5, 8, and 10, among others (Figure 3). Thus, NKTT320 can efficiently engage the iTCR and mediate vigorous human iNKT cell activation in vitro.

### 3.3. NKTT320 Promotes the Upregulation of Cytotoxic Markers in Human iNKT Cells

**Figure 4:**
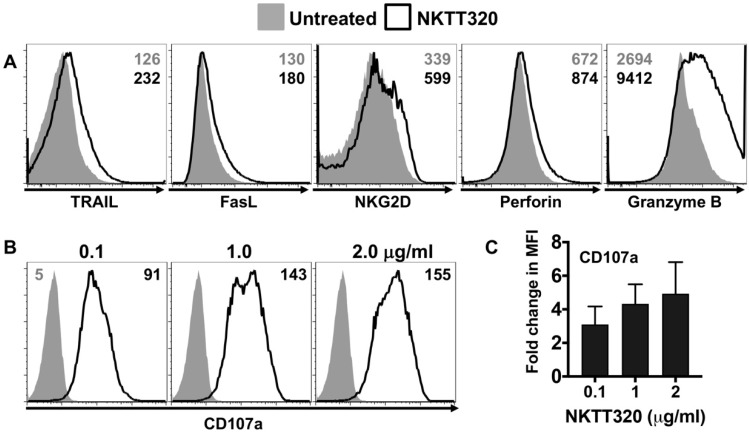
NKTT320 induces the upregulation of cytotoxic markers. (**A**) Freshly isolated human iNKTs were added to wells coated with plate-bound NKTT320 mAb (1.0 μg/mL) or left untreated. After 24 h, cells were harvested and analyzed for surface expression of NKG2D, death receptors (TRAIL and FasL), or intracellular levels of lytic molecules (perforin and granzyme (**B**)). (**B**,**C**) Human iNKTs were incubated with no stimulus or with different concentrations of plate-bound NKTT320 mAb as indicated, and analyzed for CD107a expression by flow cytometry. Data in (**A**,**B**) are representative of at least two independent experiments. Numbers in the histograms indicate MFI. Pooled data (mean ± SEM) from four independent experiments show fold increases in CD107a (**C**) expression on activated iNKTs, compared to those left untreated.

Invariant NKT cells exert potent and direct cytolytic activity via the release of perforin and granzyme [30,31], or through expression of membrane-bound receptors of the tumor necrosis factor (TNF) family, Fas ligand (FasL), and TRAIL [32,33]. Additionally, human iNKTs can mediate the direct lysis of target cells via NKG2D (cellular stress ligand receptor) engagement independent of TCR–CD1d interaction [34]. To determine whether NKTT320 promotes iNKT cell degranulation or expression of death-inducing receptors, we cultured purified human iNKTs in the presence of the immobilized mAb and measured the expression of NKG2D, TRAIL, and FasL, as well as perforin, granzyme B, and CD107a, by flow cytometric analysis (Figure 4A,B). We observed about a 2.0-fold increase in the expression of NKG2D and TRAIL, with little to no change in FasL or perforin expression. In contrast, intracellular expression of granzyme B (2.95 ± 0.55 fold) and CD107a (4.93 ± 1.87 fold) was significantly increased in the presence of plate-bound NKTT320 (Figure 4A,B). Similarly, soluble NKTT320 induced robust expression of granzyme B and CD107a, with modest to no change in the expression of death receptors or perforin. 

### 3.4. NKTT320-Stimulated Human iNKTs Transactivate Bystander Immune Cells In Vitro

**Figure 5:**
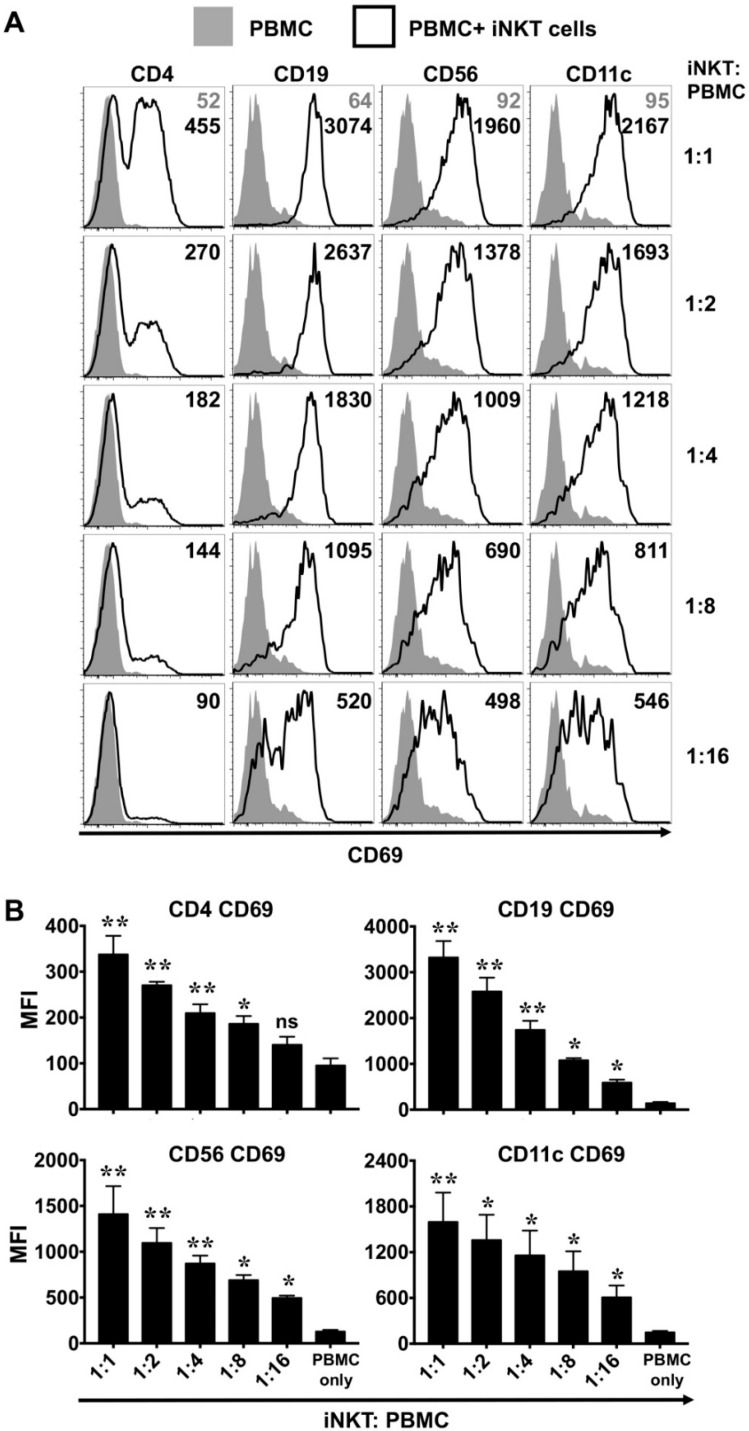
NKTT320-stimulated human iNKTs trans-activate bystander immune cells in vitro. (**A**,**B**) Freshly isolated human iNKTs were mixed with autologous PBMCs at different ratios as indicated. Control wells had PBMCs only. Cells were added to plate-bound NKTT320 mAb (1.0 μg/mL). After 24 h, cells were harvested and analyzed for CD69 by flow cytometry on different immune cells as indicated. Representative histograms from one of at least four independent experiments are shown in (**A**). Numbers in the histograms indicate MFI. (**B**) Pooled data (mean ± SEM) from 4 independent experiments showing MFI for CD69 expression on various immune cells, as indicated on the graphs. Statistical significance was determined using one-way ANOVA. For each of the analysis, all the groups were compared to control wells (PBMC only). * *p* < 0.05, ** *p* < 0.01, ns: not significant.

Once activated, iNKTs serve to mature dendritic cells (DCs) and promote the functions of NK, T, and B cells [3]. To investigate whether NKTT320 promotes iNKT cell-dependent activation of bystander lymphocytes and DCs, we cultured PBMCs with or without added purified autologous human iNKTs on plate-bound (1 μg/mL; Figure 5) or soluble (10 μg/mL; Supplementary Figure S1) NKTT320. Twenty-four hours later, cells were harvested and analyzed by flow cytometry for the expression of CD69 on various lymphoid (CD4+, CD19+, and CD56+) and myeloid (CD11c+) cell populations. In the absence of added autologous human iNKTs, we did not observe upregulation of CD69 on T (CD4+), B (CD19+), NK (CD56+), or DCs (CD11c+) within the PBMC cultures, regardless of whether they were cultured in the presence of immobilized (Figure 5) or soluble (Supplementary Figure S1A) NKTT320. However, when cultures were supplemented with iNKTs, all these immune cell populations exhibited a dramatic increase in the surface expression of CD69 (Figure 5 and Supplementary Figure S1A). Indeed, we observed that the increase in the MFI for CD69 was directly proportional to the number of iNKTs in culture, suggesting that NKTT320 was activating iNKTs, which in turn was driving activation of other immune cells present in the cultures.

### 3.5. NKTT320-Activated Human iNKTs Induce Cytokine Production and NK Cell Degranulation In Vitro

**Figure 6:**
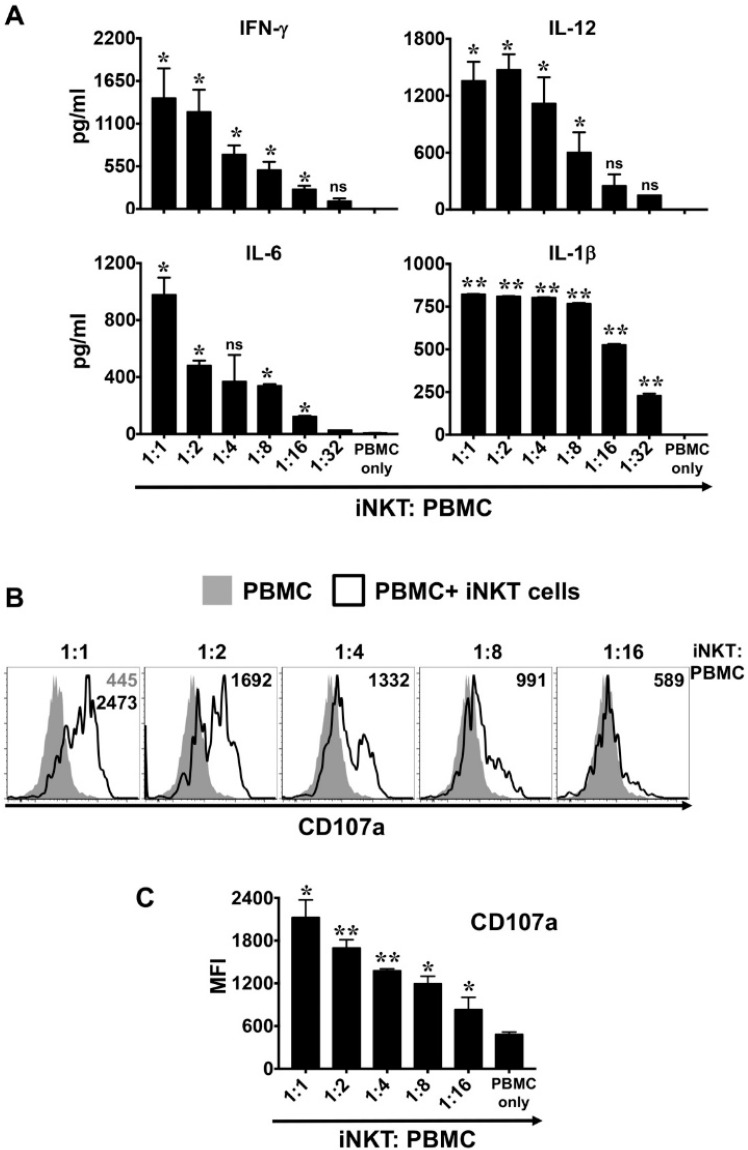
NKTT320 induces bystander cell cytokine production and NK cell degranulation in vitro. Freshly isolated human iNKT cells were mixed with autologous PBMCs at different ratios, as indicated. Control wells had PBMCs only. Cells were added to plate-bound NKTT320 mAb (1.0 μg/mL). After 24 h, cells and culture supernatants were harvested and analyzed for various cytokines by ELISA (**A**) and CD107a expression on NK (CD56 + TCRβ-) cells, respectively. Cytokine levels (mean ± SEM) in culture supernatants from three independent experiments are presented. Representative histograms (in (**B**)) are from one of three independent experiments. Numbers in the histograms indicate MFI. (**C**) Pooled data (mean ± SEM) from three independent experiments showing MFI for CD107a expression on CD56+ cells. Statistical significance in (**A**,**C**) was determined using one-way ANOVA. For each of the analysis, all the groups were compared to control wells (PBMC only). * *p* < 0.05, ** *p* < 0.01, ns: not significant.

Invariant NKTs contribute to the regulation of anti-tumor immunity via induction of cytokine production by other immune cells. To determine the effect of NKTT320 on iNKT-cell induced cytokine response of PBMCs, we co-cultured human iNKTs and autologous PBMCs on plate-bound NKTT320. Analysis of these culture supernatants revealed that NKTT320 induced robust production of Th1 cytokines, but only when iNKTs were present (Figure 6A). When compared to the results of the prior experiments, in which purified iNKTs were plated on immobilized NKTT320 in the absence of added PBMC (Figure 3), we observed that supernatants from co-cultures containing PBMCs + iNKTs exhibited much higher levels of IL-1β (produced by monocytes), IL-6 (produced by monocytes and macrophages), and IL-12 (produced by DCs). Taken together, these observations strongly suggest that these additional cytokines are not originating from iNKT cells themselves. Rather, NKTT320 mAb is activating iNKTs, which in turn are driving cytokine production by other immune cells present in the cultures. Similarly, there was no upregulation of CD107a on NK cells (Figure 6B and Supplementary Figure S1B). However, when cultures were supplemented with iNKTs, NK cells exhibited a dramatic increase in the surface expression of CD107a.

### 3.6. NKTT320 Promotes Upregulation of Co-Stimulatory and MHC II Molecules on Antigen Presenting Cells (APCs) In Vitro

**Figure 7:**
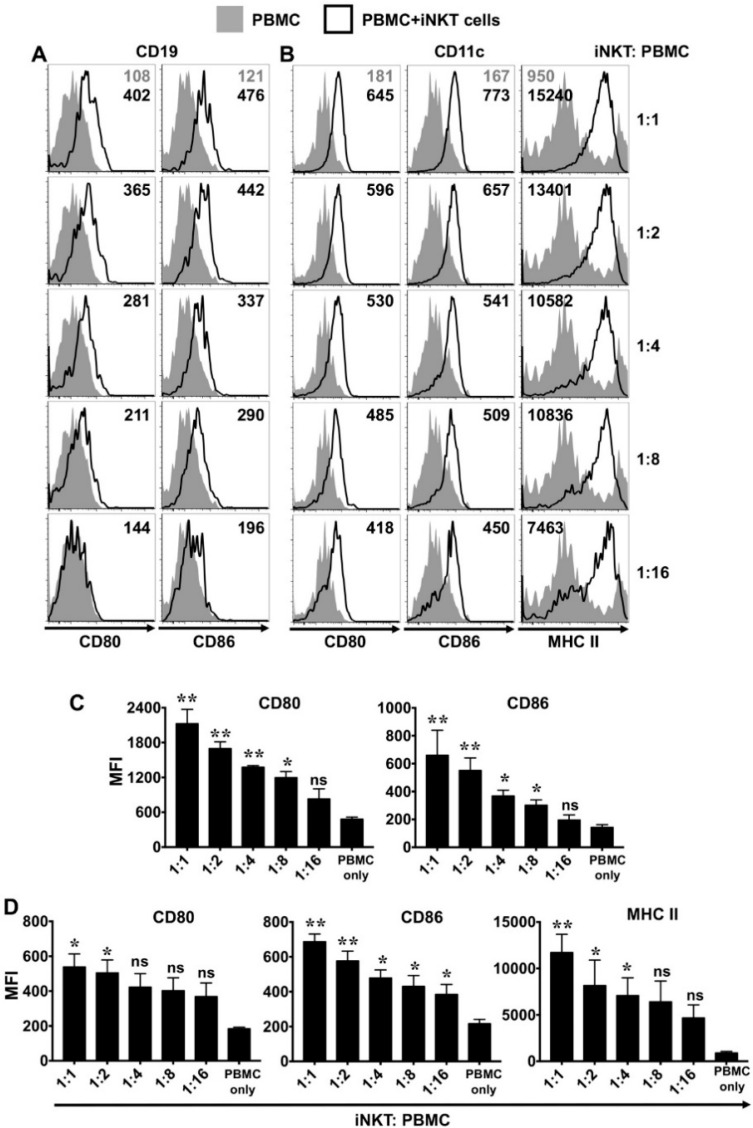
NKTT320 promotes the increased expression of co-stimulatory molecules and MHC class II (MCH II) on antigen-presenting cells. (**A**,**B**) Freshly isolated human iNKTs were mixed with autologous PBMCs at different ratios, as indicated. Control wells had PBMCs only. Cells were added to plate-bound NKTT320 mAb (1.0 μg/mL). After 24 h, cells were harvested and analyzed for surface expression of co-stimulatory molecules (CD80, CD86) and MHC II expression on CD19+ B lymphocytes (**A**) and CD11c+ myeloid cells (**B**). Representative histograms from one of three independent experiments are shown. Numbers in the histograms indicate MFI. (**C**,**D**) Pooled data (mean ± SEM) from three independent experiments show the MFI for CD80 and CD86 gated on CD19+ cells (**C**), as well as the MFI for CD80, CD86, and MHC II expression on CD11c+ cells (**D**). Statistical significance determined using one-way ANOVA. For each of the analysis, all the groups were compared to control wells (PBMC only). * *p* < 0.05, ** *p* < 0.01, ns: not significant.

To investigate whether NKTT320 promotes the activation of APCs, we cultured PBMCs with or without added iNKTs in the presence of plate-bound (Figure 7A,B) or soluble mAb (Supplementary Figure S1C,D). In the absence of added human iNKTs, PBMC plated on immobilized or soluble NKTT320 failed to upregulate co-stimulatory (CD80 and CD86) or MHCs class II molecules on APCs, such as CD19+ B and CD11c+ DCs. On the other hand, co-culture of PBMC with purified iNKTs and NKTT320 dramatically increased the expression of these molecules on both B cells and DCs (Figure 7A,B and Supplementary Figure S1C,D). Similar to CD69 expression, the increase in MFI for CD80, CD86, and MHC class II (MCH II) was directly proportional to the number of iNKTs in the culture, further supporting that NKTT320-stimulated human iNKTs facilitate the activation of APCs in the iNKT–PBMC co-cultures.

## 4. Discussion

Favorable results from mouse preclinical models and human clinical trials support development of iNKT cell-based approaches for the treatment of cancer [35,36]. Indeed, the adoptive transfer of autologous iNKT cells, along with the agonistic lipid αGC [37] or αGC-loaded DCs [38], has proven beneficial in the treatment of patients with cancer. However, iNKT cell numbers are diminished in many cancer patients, and are often accompanied by functional defects, such as decreased proliferation and poor cytokine production [5,6,7,8]. Therefore, while iNKT therapies appear well-tolerated, objective clinical response rates are transient, and occur in only a subset of patients [35,36]. To capitalize on the potential benefits of iNKT cell-based cancer therapies, it is imperative that new strategies are developed to enhance the functions of patient-derived iNKT cells. Due to limited variation within the human *CD1D* gene (which encodes CD1d), such strategies can also be applied to enhance the tumor-directed functions of allogeneic iNKT cells isolated from healthy donors. 

Several challenges exist that undermine the clinical outcome of iNKT cell-based therapies. For instance, tumor cells can dampen antitumor immune responses by inducing iNKT cell anergy and exhaustion [39]. In addition, optimal iNKT cell-mediated, anti-tumor responses generally require tumor cell expression of CD1d [21,22,23]. However, many tumors down-regulate CD1d and escape iNKT cell recognition. To overcome this critical barrier, we and others have designed and tested alternative strategies, such as chimeric antigen receptor (CAR) therapy [24] and bi-specific fusion proteins [25,26,40]. These approaches hold great potential for clinical use, as they promote robust iNKT cell activation and augment anti-tumor efficacy in a CD1d-independent manner. However, their wider therapeutic application and clinical success relies on the identification of tumor-associated antigens (TAAs) that are specifically expressed on tumors and are not present in normal, healthy cells. Furthermore, TAAs exhibit significant inconsistency in their immunogenicity, can undergo immune editing, and may be differentially expressed among patients, even when they have the same cancer type [41]. The successful clinical application of monoclonal antibodies (mAbs) against checkpoint regulators like PD1/PD-L1 and CTLA-4 has transformed the field of cancer immunotherapy [42]; however, their effect on iNKT cells remains to be fully elucidated. 

One approach for successful application of iNKTs in cancer immunotherapy could be using mAbs that directly bind to the iTCR and induce a robust iNKT cell functional response. Indeed, we demonstrate here that NKTT320, a novel humanized mAb specific for the invariant TCR of human iNKTs, stimulates robust iNKT cell activation and proliferation (Figure 1 and Figure 2) without compromising iNKT cell viability (Figure 1A). NKTT320 also upregulated granzyme and CD107a expression in human iNKTs (Figure 4B,C). Given that iNKTs primarily mediate their cytolytic activity via the exocytosis of lytic granules, our data suggest that NKTT320 is likely to promote iNKT cell-mediated direct cytotoxicity. Finally, NKTT320-induced iNKT cell activation was coupled with copious production of several Th1 cytokines, including IFN-γ, IL-2, TNF-α, IL-8, and GM-CSF (Figure 3). The functional relevance of this observation is consistent with prior findings demonstrating that activation of iNKTs results in “adjuvant effects” during antitumor immunity by activating other cytotoxic lymphocytes, mainly through a Th1 cytokine cascade. 

Once activated, iNKTs can orchestrate innate and adaptive immune responses by priming the DCs to produce IL-12, which in turn can drive the anti-tumor functions of NK and CD8+ T cells [43,44,45]. While the iNKTs themselves did not produce significant amounts of IL-12 (data not shown), it was readily detectable in the co-cultures of iNKTs and autologous PBMCs plated on immobilized NKTT320 (Figure 6A). As IL-12 is primarily produced by DCs, macrophages, and B cells [46], the increased IL-12 levels in the culture supernatant are likely due to activation of these immune cells, and not the iNKTs. In support of this notion, we observed enhanced expression of co-stimulatory molecules (CD80 and CD86) and MHC II on B cells and DCs (Figure 7). Besides IL-12, we observed significantly increased levels of IFN-γ (produced by CD8+ cells, NK cells, and the iNKTs), as well as IL-1β and IL-6 (cytokines produced by monocytes and macrophages), in co-cultures of iNKTs/PBMCs plated on immobilized NKTT320 (Figure 6A). We also observed robust upregulation of the activation marker CD69 on various lymphocytes (Figure 5) and degranulation by NK cells (Figure 6B) in co-cultures of iNKTs/PBMCs in the presence of NKTT320. Thus, NKTT320 stimulates iNKTs, which in turn activate cells of both the innate and adaptive immune system, necessary for a robust and sustained anti-tumor response. In agreement with these in vitro data, we observed that treatment of Vα24 transgenic mice (which express the human iTCR alpha chain) [47] with NKTT320 led to increased iNKT cell activation (indicated by the up-regulation of activation markers, such as CD25 and CD69), proliferation (increased BrdU incorporation), and IFN-γ production (data not shown). Altogether, these data reveal that NKTT320 effectively engages human iTCRs and induces potent iNKT cell-dependent immunostimulatory activity in vitro and in vivo. 

Consistent with our current observations, prior studies have shown that mAbs designed specifically against the iTCR of murine or human iNKTs can induce their activation or depletion. Specifically, 6B11, an mAb for human iNKTs, can be used to selectively identify, isolate, and expand human iNKTs [48]. Accordingly, 6B11 is invaluable for preclinical studies and diagnostic purposes; however, unlike NKTT320, it is not a humanized mAb, and thus there are significant challenges surrounding its use in the clinical setting. While the presence of iNKTs are favorable for host anti-tumor immunity, they are undesirable in inflammatory conditions, such as asthma and sickle cell disease (SCD). As such, NKTT120 [49,50], a humanized anti-iNKT cell mAb that selectively depletes iNKTs, has been tested and safely administered to cynomolgus macaques [50] and adults with SCD [49]. Similarly, the activating (NKT14m) and depleting (NKT14) versions of a murine iTCR-specific mAb can delay or accelerate the onset of Type 1 diabetes in NOD mice [27]. 

Collectively, these studies establish that iNKT cell functions can be effectively modulated by anti-iTCR mAbs. To that end, in a recent study it was shown that when NKT14m antibody is administered in combination with the chemotherapeutic agent cyclophosphamide to tumor-bearing mice, it significantly increases the anti-tumor response and prolongs survival [28]. In the same study, repeated injection of NKT14m did not induce iNKT cell anergy, a phenomenon that has restricted the clinical use of the iNKT cell agonist αGC [28]. In a similar manner, our data reveal that NKTT320 effectively engages the human iTCR and induces potent iNKT cell-dependent immunostimulatory activity, reinforcing the use of such antibodies to bolster iNKT cell anti-tumor activity. One potential drawback of the anti-iTCR, mAb-based cancer therapy could be the uncontrolled activation of iNKTs, leading to severe adverse effects, including cytokine release syndrome. However, several phase I clinical trials with αGC-loaded immature or mature DCs have led to clinically relevant anti-tumor responses with minor systemic side effects [51,52,53,54,55]. Given that iNKTs are relatively rare, both in circulation and within the tumor, increasing their numbers and function (such as an increase in IFN-γ production and upregulation of granzyme B) should be safe. Indeed, in a clinical trial [53], 100-fold expansion of circulating iNKTs resulted in no more than grade I toxicity. 

In conclusion, to be most effective, cancer immunotherapies must include combination strategies that engage and activate innate as well as adaptive immunity by simultaneously targeting multiple components that can augment each other’s activity. As iNKTs not only kill tumor cells themselves, but also link the innate and adaptive immune systems and can reinvigorate exhausted immune cells in the tumor microenvironment, they make excellent candidates for inclusion in cell-based therapies for cancer. Our findings broaden the scope of iNKTs for clinical application by providing a viable strategy to modulate the host’s immunity against various cancers. 

## Supplementary Materials

The following are available online at https://www.mdpi.com/1422-0067/21/12/4317/s1.
