# Supplementary material for: Cancer Immunotherapeutic Potential of NKTT320, a Novel, Invariant, Natural Killer T Cell-Activating, Humanized Monoclonal Antibody
Source: Int J Mol Sci. 2020 Jun 17;21(12):4317. doi: 10.3390/ijms21124317 (PMC7352964; doi:10.3390/ijms21124317)
Supplement: Supplementary file 1 [file ijms-21-04317-s001.pdf]

Sup Fig. 1

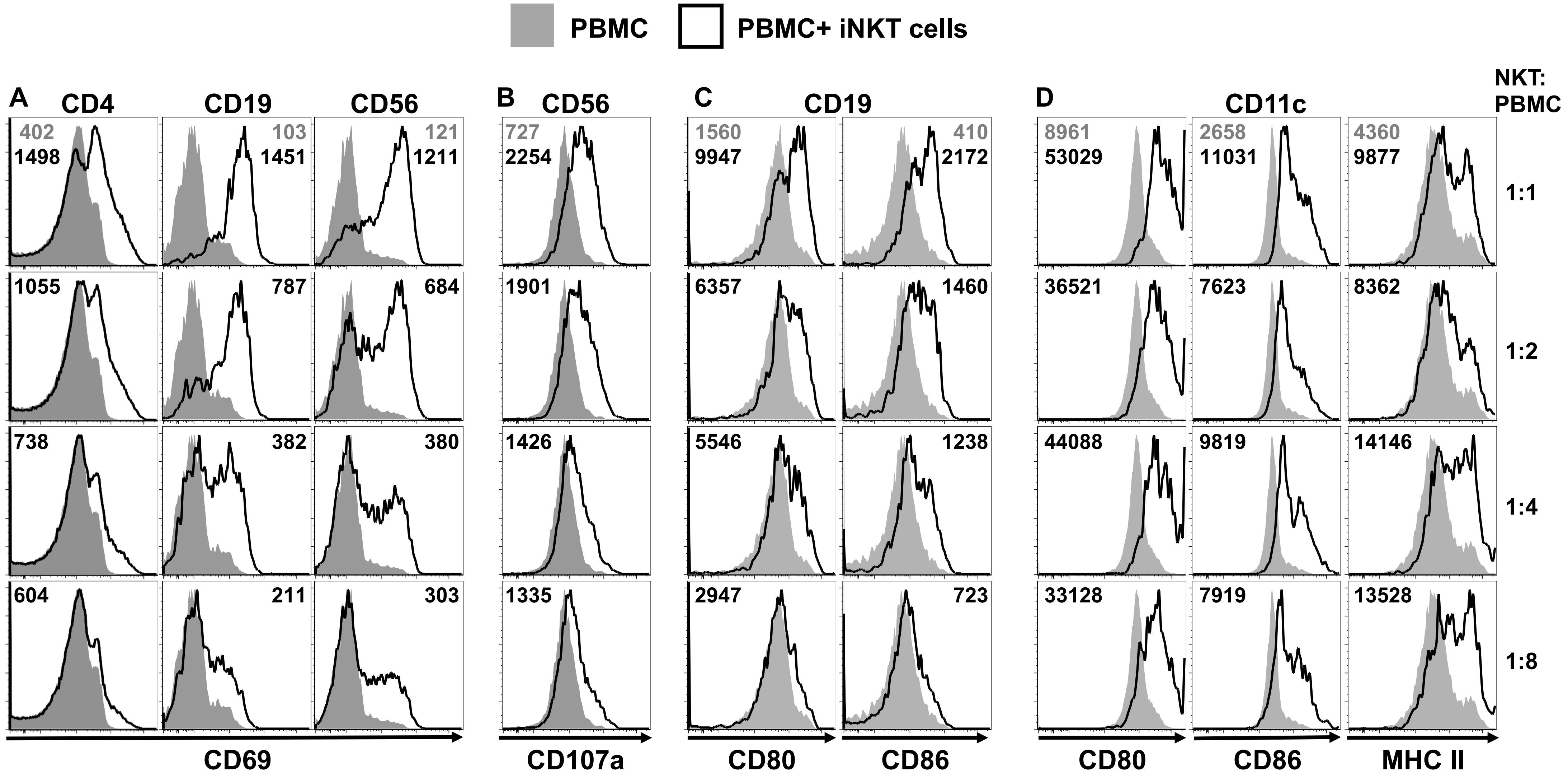
PBMC
PBMC+ iNKT cells
A
CD4
CD19
CD56
B
CD56
C
CD19
D
CD11c
NKT:
PBMC
402
1498
103
1451
121
1211
727
2254
1560
9947
410
2172
8961
53029
2658
11031
4360
9877
1:1
1055
787
684
1901
6357
1460
36521
7623
8362
1:2
738
382
380
1426
5546
1238
44088
9819
14146
1:4
604
211
303
1335
2947
723
33128
7919
13528
1:8
CD69
CD107a
CD80
CD86
CD80
CD86
MHC II

Supplemental Figure Legends

Sup Figure 1. Soluble NKTT320 promotes human NKT cell-mediated transactivation of bystander immune cells. Freshly isolated human iNKTs were co-cultured with autologous PBMCs at different ratios as indicated. Control wells had PBMCs alone. Both control and experimental wells received soluble NKTT320 mAb (10 μg/ml). After 24 h, cells were harvested and analyzed for (A) CD69 and (B) CD107a expression on different immune cells by flow cytometry. (C, D) Surface expression of co-stimulatory molecules (CD80, CD86) and MHC II expression on B (CD19+) and myeloid cells (CD11c+) were assessed by flow cytometry. Representative histograms (with MFI) from one of three independent experiments are shown. Numbers in the histograms indicate MFI.
